# Challenges in Food Chemistry

**DOI:** 10.3389/fnut.2015.00011

**Published:** 2015-04-27

**Authors:** Michael Rychlik

**Affiliations:** ^1^Technische Universität München, Freising, Germany

**Keywords:** authenticity, globalization, modified mycotoxins, molecular sensory science, non-communicable diseases

## Background

### Changes in developed societies

In the course of the twenty-first century, the developed societies have to face various challenges, which are complemented with challenges to all scientific disciplines dealing with food and nutrition. First of all, demographic changes will lead to an increasing percentage of the elderly, who require foods different to those of other parts of the population. As sensory perception is altered when we grow older and often the food intake decreases, there is the need for foods with adjusted sensorial properties and particularly high in micronutrients. For instance, a deficiency in vitamin B_12_ is likely to occur as the hematologic signs of the deficiency are reported to be masked by food supplementation with folic acid.

No less important for our society is the increasing incidence of lifestyle diseases or non-communicable diseases (NCD) such as cancer, cardiovascular disease, diabetes, and chronic respiratory diseases. Among lifestyle, the individual composition of the diet holds a prominent place, e.g., 30% of all cancer casualties are supposed to be due to nutrition. NCD will affect increasingly the societies and, for their prevention, new foods and new nutrition habits will play a crucial role.

### Global markets

Food trading, more than ever, has reached global dimensions. As transport costs or trade restrictions have become less relevant, nearly all foods now may come from all over the world, particularly from these parts, where they can be produced cheaply. However, the product flows often lack transparency and, therefore, consumers increasingly demand regional or authentic foods. For these, they are willing to pay a higher price. This leads regularly to food fraud and the consumer increasingly requires proofs of authenticity.

Along with globalization of markets, the issue of food safety often occurs as food regulations and standards of food production are highly diverse on the international level. Although each country has the same requirements and regulations for imported foods as for domestic foods, control intensity of foods often depends on the origin. For example, pistachios are more frequently controlled for aflatoxins if they originate from Iran or other Central Asian countries than from the USA. This also points to the need for knowing and verifying the origin of foods.

### Multidisciplinary food chemistry

One of the main tasks of food chemistry is to understand the molecular basis of reactions in foods. To achieve this, food chemists analyze foods by using chemical, biochemical, and molecular biological methods along with high-performance instrumental equipment. In this way, they elucidate mechanisms of formation and degradation of food ingredients, identify valuable as well as undesirable compounds, and further establish structure–activity as well as dose–action relationships. This allows to understand the chemistry of processes and to establish the chemical basis for technological innovation.

However, the key to master the challenges in food chemistry is multidisciplinary by cooperating with nutritionists, toxicologists, nutritional medics, microbiologists (c.f. the microbiome), plant and animal scientists, and last but not least, food technologists.

## Future Priority Areas

### Design of new foods and new food ingredients

The demographically changing population and the dramatic increase in life style diseases more than ever require the identification and quantification of bioactive compounds. With this knowledge, beneficial compounds in foods may be enhanced, e.g., by biofortification or by changes in technological processes. Moreover, new technofunctional compounds such as nanoparticles open avenues to foods with new properties, e.g., stabilizing emulsions by titanium dioxide. However, in parallel with their development, thorough risk assessment of the new additives has to keep pace.

### Authentic foods

Authenticity of foods generally is related to one or more of the following attributes: geographic origin, type of agricultural production, species and kind of raw materials, or certain process qualities such as sustainability or ecologic foot print.

Regularly uncovered scandals of food adulteration, the most recent major one being the European horse meat fraud in 2013, underline the sensitivity of consumers to this issue. Apart from meat, foods often being adulterated are olive oil, fish, organic foods, spices, tea, cocoa, coffee, and nuts.

In the recent years, there has been tremendous progress in high resolution methods to elucidate the molecular fingerprint of foods. On the genetic scale, apart from classical polymerase chain reaction, new developments of isothermal amplifications or next generation sequencing will enable more accurate identification of species.

On the protein level, specific biomarker peptides can be used, e.g., for species differentiation of meat after tryptic digestion of myosine by LC-MS/MS (1).

For a fingerprint of metabolites, the new methods of non-targeted and targeted metabolomics already allow a specific authentification. When regarding wine, not only information about grape variety, terroir, vintage, and enological practices are accessible by Fourier transform ion cyclotron mass spectrometry (FT/ICR-MS) but also the species and the origin of the oaks used to assemble the barrels in which the vine was stored (2). A further method for profiling is ICP-MS of rare earth elements (3), which allows to differentiate various foods including wine or coffee. All these new methodologies generate “big data,” from which the relevant information is only accessible when applying novel bioinformatics approaches.

### Safe foods

Apart from microbiological decay and food-borne infections, contaminants endanger the safety of all links in the whole food chain. The recent discoveries of process contaminants range from simple molecules such as acrylamide, furan, benzene, styrene to more complex compounds such as 3-monochloropropane-1,2-diol (MCPD) esters. An end of new discoveries cannot be foreseen yet and we may assume that the sum of all these contaminants has a significant impact on life style diseases such as cancer. Further, new contaminants arise from packaging materials such as mineral oil saturated hydrocarbons (MOSH) or mineral oil aromatic hydrocarbons (MOAH), and pollutants from the environment such as the polyfluorinated alkyl substances (PFAS). Moreover, the historic toxin arsenic is more relevant than ever as rice and rice products are often contaminated and the mechanisms of arsenic carcinogenicity are still under controversial discussion. Another field with ongoing attention is the mycotoxin issue, as the so-called “masked” forms required a new definition, which was found with “modified” mycotoxins including all sorts of biological, chemical modifications, and matrix associations (4). This concept of differentiating between the originally biosynthesized metabolite by one species and any metabolization or chemical modification by successional processes may also be applied to other bioactive compounds such as phenolics or vitamins. Thus, the products formed by glucuronidation in mammals or by oxidative degradation analogously may be termed “modified (1^st^ level), biologically modified (2^nd^ level), conjugated (3^rd^ level) by animals (4^th^ level)” or “modified (1^st^ level), chemically modified (2^nd^ level), thermally formed (3^rd^ level),” respectively.

Although a comprehensive definition has been found, we are still far away from a satisfying risk assessment of modified mycotoxins. However, they have to be considered as the modified toxin deoxynivalenol-3-glucoside has been shown to be cleaved to deoxynivalenol (DON) in the gut and thus contributes to the toxicity of the latter without being regulated yet. It appears only as the tip of the iceberg as only few modified mycotoxins have been identified, and not to mention the lack of toxicological data for those few we know. There is an urgent need for accurate analytical methods to obtain reliable exposure data as well as toxicological data.

Apart from these “new” contaminants, almost completely missing is an assessment of combination effects of toxins, be it within one group of compounds or spanning various structural groups. The current concept for assessing combinatory effects is that of cumulative assessment groups (CAGs) (5), which, e.g., assesses the cumulative potency-corrected dose of acute reference doses (ARfD) for pesticides showing the same mode of toxic action. However, this approach is still preliminary and lacks comprehensive confirmation as unpredictable metabolomic profiles of combined pesticide actions (6) point to the need for further research.

### Tasty and odorous foods

Other important quality parameters of foods are odor and taste and in this regard there are further challenges for molecular sensory science as a discipline of food chemistry. Whereas it is likely that the major key food odorants already have been unraveled, there are still further taste active and modulating compounds to be identified. Although many odorants are known, their precursors and their mode of generation in some cases still are open. With this knowledge, it will be possible to better select for cultivars or processes to improve aroma quality. Moreover, consumer acceptance of some foods is still low and their formulation needs to be improved. In particular, in infant foods, odor and perception of which are intended to be similar to that of breast milk, are another focus of flavor research (7).

Besides odor activity, bioactivity of odorants and tastants is also being investigated, either of the endogenous odorants or of their metabolites.

For tastants, matrix interactions also are important, e.g., for sodium. Recent studies are directed toward sodium reduction in cereal products at equal salty sensation as the average consumption of sodium is significantly higher than the recommended maximum.

Apart from odorants or tastants, other compounds and matrix effects influencing the psychochemistry of our eating behavior have been located. In the new field of neurotrition, e.g., food-specific effects on satiety have been elucidated, e.g., the satiable effect of olive oil (8) or the unstoppable appetite for potato chips has been ascribed to a combination of macronutrients (9). However, the molecular principle and processes still have to be found.

## Concluding Remarks

The twenty-first century holds major challenges in store for food chemists. Quality of foods has to be secured and improved to meet the needs of growing populations in developing countries and emerging economies as well as of the aging population in developed societies. However, expectable progress in instrumentation, online bioactivity-guided screening, and improved molecular understanding will help scientists to improve important aspects of food quality such as authenticity, safety, functionality, and savoriness.

## Conflict of Interest Statement

The author declares that the research was conducted in the absence of any commercial or financial relationships that could be construed as a potential conflict of interest.
